# Supplementary material for: Impact of natural disasters on consumer behavior: Case of the 2017 El Niño phenomenon in Peru
Source: PLoS One. 2021 Jan 28;16(1):e0244409. doi: 10.1371/journal.pone.0244409 (PMC7842981; doi:10.1371/journal.pone.0244409)
Supplement: S1 File — (PDF) [file pone.0244409.s001.pdf]

S1 File

Demographics information and known bias in our dataset

As of July 2019, the Peruvian National Institute of Statistics and Informatics reported a population of approximately 33 million, of which 22 million represent an economically active population [1]. In the capital city, Lima there are 8.5 million inhabitants. As a reference, the Peruvian economy is one of the world's fastest-growing economies as of 2000. Like the rest of Latin America, Peru has a fast-growing debit/credit card market, and 39% of the population owns a bank account, while 29% of the population owns a credit/debit card [2]. We estimated individuals' socio-economic class in our dataset by relying on the consumption captured by the average monthly purchase (AMP) P_i [3] (11).

$$P_i = \frac{\sum_{t \in T} P_i(t)}{|T|_i} \tag{11}$$

Here $P_i(t)$ is the total number of purchases by individual i in a given month t, and $|T|_i$ is the number of months in which individual i made at least one purchase. It should be noted that we considered only individuals with more than \$30 of purchases in all months. We then computed the normalized cumulative distribution function $C(f)$ as a function of the fraction f of people (12).

$$C(f) = \frac{1}{\sum_i P_i} \sum_f P_{i.} \tag{12}$$

Based on the cumulative AMP, we split the population into nine economic classes (see Fig. S1 **a**). Subsequently, we derived a set of demographics from the individuals in our dataset S1 such as the social class distribution, population pyramid, and gender imbalance. The population pyramid of our dataset appeared to be in accordance with the population pyramid of the Peruvian population [4]. However, our dataset appeared to have a bias toward the male population because we observed a gender imbalance. This was also observed in a study by [17] that used the same type of dataset for Mexico instead of Peru.

Finally, we computed the GINI coefficient G (13) based on the (AMP) and found coefficient values ranging from G = 0.60 to 0.66 instead of $G = 0.433$, as provided by the World Bank [5]. This substantial difference between the two coefficients may be due to two phenomena. First, we may have had an overrepresentation of upper-class individuals in our dataset that may have biased some of our results [6]. Second, the GINI coefficient we computed here is based on the AMP only; that is, it is based on people's spending instead of taking into account their income plus the benefit received from social programs [7].

$$G = \frac{\sum_{i=1}^{n} (2i - n - 1) P_i}{n \sum_{i=1}^{n} P_i} \tag{13}$$

District level analysis of the Kullback-Leibler divergence

In Fig. S2, we present the daily evolution of the KLD per district of the greater area of Lima (Peru) over the two years of our dataset. This figure illustrates that the KLD remained neutral (at approximately zero), which signifies that the spending distribution of the area remained consistent with the average spending behavior of the district. In contrast, when a divergence appears, it signifies that the spending distribution shifted from its normal behavior. Fig. 2 also demonstrates that the February 2017 events impacted most of Lima's districts, and a spike on February 20 can be clearly observed.

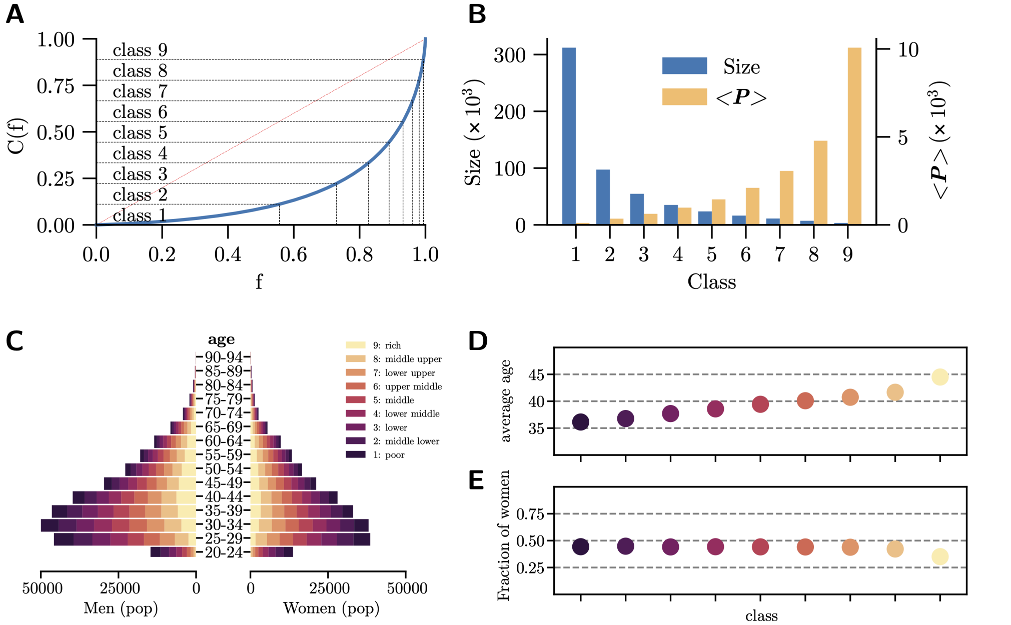

Fig S1. Demographic characteristics of dataset. a) Social class distribution. b) Average AMP $\langle P \rangle$ in each social class and the number of people in each social class. c) Age pyramid for males and females. d) Average age in each social class. e) Fraction of females in each social class.

A subset of districts was also impacted twice by the February and March events, including the district of *Los Olivos*, *Magdalena del Mar*, and *San Miguel*. The February event observed was partially due to flooding caused by the *Rimac* and *Huaycoloro* rivers affecting the district of *San Juan de Lurigancho*. Second, during the March event, there was a substantial increase in consumption due to low supply and the overvaluation of necessities, such as mineral water, rice, and meats. Among the 42 districts of Lima, official reports [8] established that the most affected districts were the districts of *Chaclacayo*, *San Juan de Lurigancho*, *Cieneguilla*, *Punta Hermosa*, *Pucusana* and *Rimac* (see Fig. S2). In Fig. S2, the KLD measure displays a spike of activity in the reported districts during the events. This spike is a clear indication that a sudden shift in the consumption pattern occurred during the events. However, at the macroscopic level, the change in consumption behavior did not seem to persist for a long time after the events.

Causality analysis of ENSO on individual purchasing behavior

Fig. S3 displays the causal impact of El Niño on the 42 districts of Lima metropolitan area during March 2017. As in the experiment depicted in Fig. 3, we used the Callao series as the control. The negatively impacted districts were as follows *Pucusana, Carabayllo, Lurigancho, Los Olivos, Ancon, Chorrillos, Santa Rosa, San Bartolo, La Molina, Jesus Maria Surquillo, Chaclacayo, Santa Maria del Mar, Villa el Salvador, Punta Hermosa, Lince* and Lurin. In contrast, some districts such as *Lima, Pachacamac, San Isidro, San Borja, El Agustino, Independencia, San Juan Miraflores*, and *Miraflores* experienced a positive impact. Finally, the remaining districts experienced a neutral impact.

With regard to the variation in the impact of El Niño between February and March 2017 (see Fig. 3), there was a decrease in the number of negatively impacted districts from 20 in February to 17 in March (see Fig. S3 **a**). The same pattern occurred with

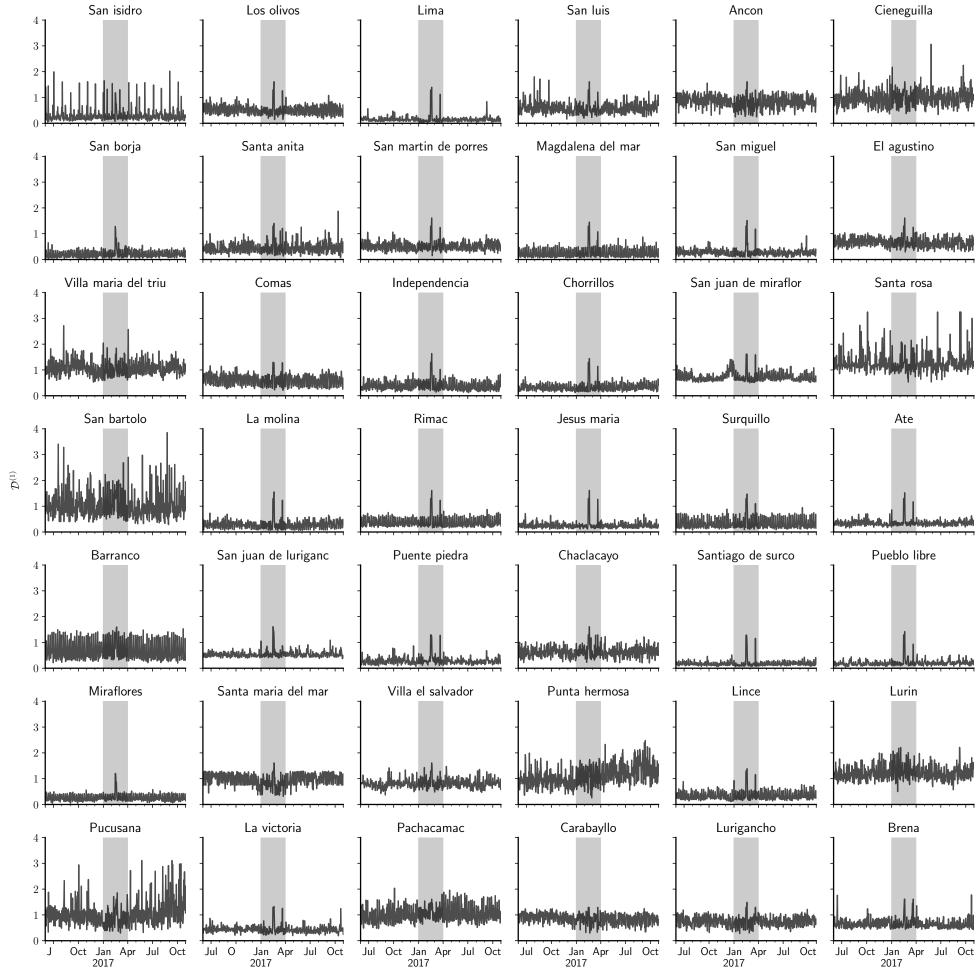

Fig S2. KLD of various districts of Lima.

positively impacted districts with a reduction from 11 in February to 8 in March (see Fig. S3 **b**). Finally, we note that more districts became neutral from: 12 in February and 18 in March (see Fig. S3 **c**).

k-core decomposition dynamic of the transaction graph

In Fig. S4 we consider the k-core decomposition [9] of the transaction graph to explore the graph evolution, and how many transactions are distributed in each of the k-shells of the transaction graph. In Fig. S4 **a** we considered each time slice of the transaction graph $\mathbf{G}_t$ at time t and compared it with the shell number of a node u at $t + \Delta t$ (where $\Delta t = 1$ day). The fact that the figure is not symmetric is an indication that when a node steps down from its k-shell position, it goes down many steps, on the contrary whenever a node enhances its k-shell position, it climbs up only one step at a time. In Fig. S4 **b**, we see how the number of transactions is distributed into each of the k-shells (the sum of the in-weights of all nodes that belong to the k-shell k).

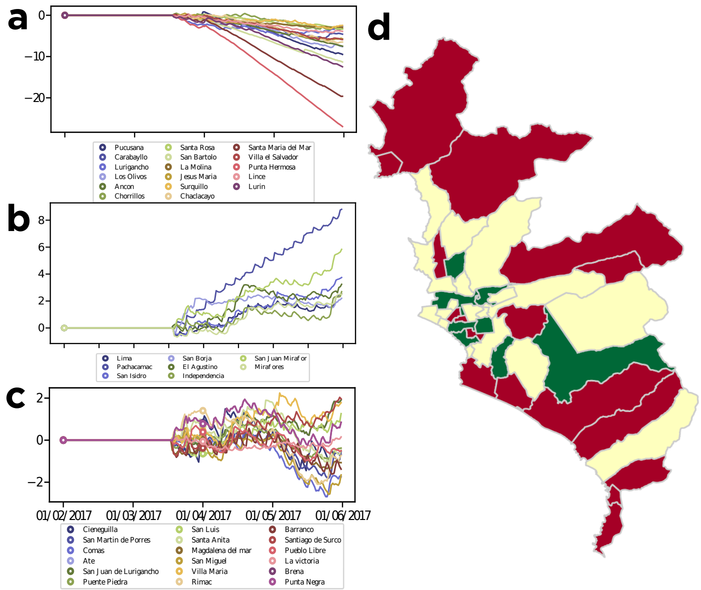

Fig S3. Causal impact at the district level for March 2017. a) List of districts with a negative impact. b) List of districts with a positive impact. c) List of districts with a neutral impact. d) Map of Lima showing the districts with a negative (red), positive (green) and neutral (yellow) impact.

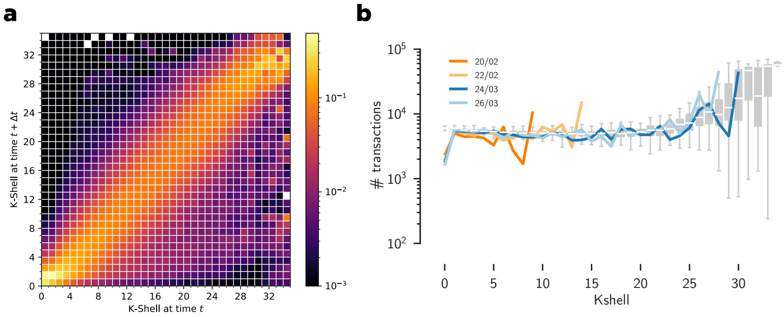

Fig S4. The Cores decomposition dynamic of the transaction graph. a) Evolution of the k-shell overtime. b) Distribution of the number of transaction as function of its k-shell.

References

1. Censos Nacionales 2017: XII de Población, VII de Vivienda y III de Comunidades Indígenas;. https://www.inei.gob.pe/media/MenuRecursivo/publicaciones_digitales/Est/Lib1544/.

2. Computop's Payment & e-Commerce Report Latin America; 2017. https://www.computop.com/fileadmin/user_upload/Downloads_Content/deutsch/CountryReports/CountryGuide_Latin_America.pdf.

3. Leo Y, Karsai M, Sarraute C, Fleury E. Correlations of consumption patterns in social-economic networks. In: 2016 IEEE/ACM International Conference on Advances in Social Networks Analysis and Mining (ASONAM). IEEE; 2016. p. 493–500.

4. Population Pyramids of Peru in 2019;. https://www.populationpyramid.net/peru/2019/.

5. GINI Peru;. https://data.worldbank.org/indicator/SI.POV.GINI?locations=PE.

6. Yamada G, Castro JF, Oviedo N, et al. Revisitando el coeficiente de Gini en el Perú: el rol de las políticas públicas en la evolución de la desigualdad; 2016.

7. Yamada G, Castro JF, Bacigalupo J, et al. Desigualdad monetaria en un contexto de rápido crecimiento económico: El caso reciente del Perú. Revista Estudios Económicos. 2012;24:65–77.

8. Informe Técnico Extraordinario 001-2017/ENFENEL NIÑO COSTERO 2017; 2017. http://www.imarpe.pe/imarpe/archivos/informes/imarpe_inftco_informe__tecnico_extraordinario_001_2017.pdf.

9. Batagelj V, Zaveršnik M. Fast algorithms for determining (generalized) core groups in social networks. Advances in Data Analysis and Classification. 2011;5(2):129–145. doi:10.1007/s11634-010-0079-y.
