# Supplementary material for: Impact of natural disasters on consumer behavior: Case of the 2017 El Niño phenomenon in Peru
Source: PLoS One. 2021 Jan 28;16(1):e0244409. doi: 10.1371/journal.pone.0244409 (PMC7842981; doi:10.1371/journal.pone.0244409)
Supplement: Attachment — Submitted filename: Response_to_Reviews.pdf [file pone.0244409.s002.pdf]

Reply to Editors PLOS ONE:

Impact of natural disasters on consumer behavior: case of the 2017 El Niño phenomenon in Peru

Hugo Alatrista-Salas, Vincent Gauthier
Miguel Nunez-del-Prado, Monique Becker

November 19, 2020

Dear chairs of the Plos One. We are most grateful for the comments on our work, which have helped us to improve the quality of our paper. Needless to say, we have carefully considered each comment and we have accordingly introduced changes in the paper, detailed below.

Please note that the reviewers' comments are typed in italics, while our own reply or explanation remains in regular style.

Reply to the editor

We thank the editor for their comments. The following paragraphs answer each one of them.

"Please ensure that your manuscript meets PLOS ONE's style requirements, including those for file naming."

The document follows PLOS ONE's style requirements.

"In your Data Availability statement, you have not specified where the minimal data set underlying the results described in your manuscript can be found. PLOS defines a study's minimal data set as the underlying data used to reach the conclusions drawn in the manuscript and any additional data required to replicate the reported study findings in their entirety. All PLOS journals require that the minimal data set be made fully available."

As we described in the Cover Letter, the dataset used for experiments was obtained from a Peruvian private financial entity (BBVA), which was provided to us in the context of a long-standing collaboration between the Universidad del Pacífico and the financial entity through a specific multi-disciplinary research agreement. Sharing the raw version of this dataset can potentially breach the privacy of the bank's customers. For this reason, we share a dataset derived from the used dataset, but that will not compromise the privacy of the BBVA customers. Moreover, it would enable the reviewer to reproduce our study and help anybody who aims to understand the consumption behavior at the country level.

The dataset we provide contains the following data:

- The consumption data aggregated by districts to enable replication of our study
- The transaction graphs datasets we used in this paper.

All the data are available at https://doi.org/10.7910/DVN/LYXBGR. It is worth to note that the dataset we provide will be shared under the CC0 1.0 Universal (CC0 1.0) Public Domain Dedication and can be cited as follows.

Miguel Nunez-del-Prado; Hugo Alatrista-Salas; Vincent Gauthier. *Impact of natural disasters on consumer behavior: case of the 2017 El Nino phenomenon in Peru.* 2020. Available from https://doi.org/10.7910/DVN/LYXBGR

```
@data{DVN/LYXBGR_2020,
author = {Miguel Nunez-del-Prado and Hugo Alatrista-Salas
    and Vincent Gauthier},
publisher = {Harvard Dataverse},
title = {{Impact of natural disasters on consumer
    behavior: case of the 2017 El Nino phenomenon in Peru}},
UNF = {UNF:6:yE3zv0RxWYrVcbaPE2jAMA==},
year = {2020},
version = {V2},
doi = {10.7910/DVN/LYXBGR},
url = {https://doi.org/10.7910/DVN/LYXBGR}
}
```

"We note that Figures 1, 2, 5, S3 in your submission contain map images which may be copyrighted. All PLOS content is published under the Creative Commons Attribution License (CC BY 4.0), which means that the manuscript, images, and Supporting Information files will be freely available online, and any third party is permitted to access, download, copy, distribute, and use these materials in any way, even commercially, with proper attribution."

Figures 1 and 2 were removed and cited using an URL. The rest of the figures were built by ourselves and can be shared under the Creative Commons Attribution License (CCAL) CC BY 4.0.

Reply to reviewer 1

"It is a well-organized paper. It covers understudied areas of disaster resiliency. The findings are thoroughly discussed and conclusions were generated based on findings. Your paper is good to publish."

We thanks the reviewer for his comment.

Reply to reviewer 2

"El Nino Southern Oscillation (ENSO) is a climatic phenomenon consisting of a temperature increase in the equatorial Pacific area. At the end of 2016 and in early 2017, ENSO had an abrupt change leading to heavy rains and floods. It is possible to see higher frequency of such atypical phenomenon due to climate change in the future. However, it remains unclear how these weather anomaly affects the consumption behavior of people as well as the resilience of retail structures."

"This paper contributes to the literature by documenting how consumption behaviors, from both macro and micro level, respond to the weather extreme events. The findings are useful to policy design that aims to mitigate the negative impacts of climatic events and improve the effectiveness of recovery efforts."

We thanks the reviewer for his comment.

"This paper use the Kullback-Leibler divergence (KLD) method to capture an anomalous change in the purchase distribution of the population (Figure

3&4). It is not straightforward enough that these changes of consumption patterns are results of ENSO. Since the El Nino is characterized by heavy rains and floods, it is useful to replicate the current figures but replace the vertical axis with rainfall or floods. We expect to see similar patterns of rainfall as in consumption. "

We thank the reviewer for his pertinent remark. However, we are not trying to show a correlation between the amount of rainfall and the consumption behavior, but we aim to show that the damages and disturbances engendered by extreme rainfalls have induced changes in the consumption behavior. Nonetheless, we updated the Figure Fig. 4 to show the cumulative amount of rainfall during the period of observation. In Fig. 4 we show the cumulative rainfall over the water catchment of Lima and the cumulative rainfall over the greater area of Lima. The data used in this figure were obtained through the Center for Hydrometeorology and Remote Sensing (CHRS) Data Portal [1]. We also updated main text in the section relative to Fig. 4.

"In addition, it is also useful to replicate the analysis using data from years without El Nino costero. We expect to see no previous patterns as in years with El Nino costero."

We agree with the reviewer about the utility to replicate the analysis using data from years without El Nino costero. Nonetheless, we do not have data about the same period from February to March in other years before or after 2017.

"Also, it is not clear whether the seasonal pattern as well as time trends of consumption are adjusted."

In the work we presented in the paper, we did not remove the seasonal trend neither adjusted consumption. The removal of the seasonal trend is easy to perform on time series data such as in [2]. However in our cases each record in our data (a purchase) is associated with a certain category (as shown in Fig. 3C and Fig. 3D), meaning that we would have to remove the seasonal trend for each of the category present in our dataset (around 230 in our case). Due to the sparsity of certain categories (in space and time), this operation would potentially be difficult to perform. Moreover, this step would introduce bias toward some categories and bias our computation of the KLD for instance. In order to clarify this point, we added in the paper a

statement saying that we did not remove the seasonal trend neither adjusted consumption.

"In the section of Causality Analysis of the ENSO, this paper discovered three modes: districts were negatively impacted, districts that continued to function as usual, and districts that experienced an increase in purchases. More discussions are needed to explain why the impacts in different regions can be positive impact, negative and neutral? How are these findings connected with rainfall or floods due to the ENSO. Answering these questions help disentangle the impacts from the ENSO instead of other economic confounders."

We thank the reviewer for his comment. We added a paragraph to clarify this point.

> To better understand the causal effects, we query to an official report issued by Instituto Nacional de Defensa Civil (INDECI), the Peruvian organism in charge of Disaster Risk Management, on February and March 2017 heavy rainfalls were recorded in several Lima districts, including San Juan de Lurigancho, Lurigancho-Chosica, Chaclacayo, Pachacamac, and Comas. These rains caused numerous floods due to the overflow of rivers Huaicoloro, Lurín, and Chillón. The INDECI report also mentions that many businesses were closed during the period, and the inhabitants of these areas were forced to make their purchases in other districts to buy food and other needs. So, these districts show a negative impact. We also hypothesize that the districts with a positive impact have received an influx of purchases from the most impacted districts.

"Third, since the data contain information social-economic factors, it would be interesting to separately estimate the impacts for cohorts with different social-economic factors. For example, does the consumption behaviors respond differently to the weather events for the male and female? How does the weather extremes affect the younger and older cohorts differently?"

We thank the reviewer for this suggestion. Indeed, we haven't fully explored how the consumption behavior has responded according to the gender. Nonetheless we show in figure 1 (of this document) the general imbalance between men and women in our dataset, already highlighted in the

supporting information of this paper. We show in Fig. 1 only slight variation of the gender balance did happen during the week of 02-13 (and 03-13) which corresponds roughly to the period of events. However a deeper study is needed to explore further if this slight variation is significant. We added the following comment in the discussion section:

> Moreover, lots remains to be learned on how we react as human and as a society during crisis such as extreme climatic events, this field remains open for further inquires. We think for instance that it would be interesting to separately estimate the impacts for cohorts with different social-economic factors; or if a person is impacted differently based on his/her gender.

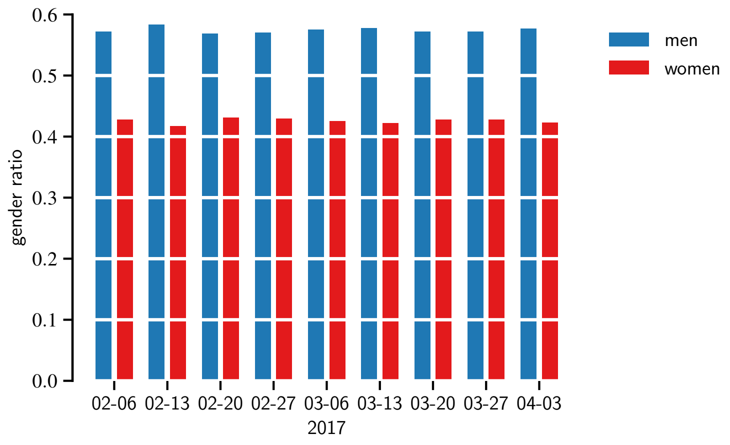

Figure 1: Evolution of the gender ratio in time. We show here the normalized number of transactions made by each gender aggregated over one week. We plot on the abscissa the date of the beginning of each week.

References

[1] Nguyen P, Shearer EJ, Tran H, Ombadi M, Hayatbini N, Palacios T, et al. The CHRS Data Portal, an easily accessible public repository for PERSIANN global satellite precipitation data. Scientific Data. 2019;6(1):180296. doi:10.1038/sdata.2018.296.

[2] Cleveland RB, Cleveland WS, McRae JE, Terpenning I. STL: A Seasonal-Trend Decomposition Procedure Based on Loess (with Discussion). Journal of Official Statistics. 1990;6:3–73.
